# Quantification of T-Cell and B-Cell Replication History in Aging, Immunodeficiency, and Newborn Screening

**DOI:** 10.3389/fimmu.2019.02084

**Published:** 2019-08-29

**Authors:** Ruud H. J. Verstegen, Pei M. Aui, Eliza Watson, Samuel De Jong, Sophinus J. W. Bartol, Julian J. Bosco, Paul U. Cameron, Robert G. Stirling, Esther de Vries, Jacques J. M. van Dongen, Menno C. van Zelm

**Affiliations:** ^1^Department of Immunology, Erasmus MC, University Medical Centre, Rotterdam, Netherlands; ^2^Division of Rheumatology, Department of Paediatrics, The Hospital for Sick Children, Toronto, ON, Canada; ^3^Division of Clinical Pharmacology and Toxicology, Department of Paediatrics, The Hospital for Sick Children, Toronto, ON, Canada; ^4^Department of Immunology and Pathology, Monash University, Melbourne, VIC, Australia; ^5^The Jeffrey Modell Diagnostic and Research Centre for Primary Immunodeficiencies, Melbourne, VIC, Australia; ^6^Department of Allergy, Immunology and Respiratory Medicine, The Alfred Hospital, Melbourne, VIC, Australia; ^7^Tranzo, Scientific Center for Care and Welfare, Tilburg University, Tilburg, Netherlands; ^8^Laboratory for Medical Microbiology and Immunology, Elisabeth-TweeSteden Hospital, Tilburg, Netherlands; ^9^Department of Immunohematology and Blood Transfusion, Leiden University Medical Centre, Leiden, Netherlands

**Keywords:** T-cell replication, TREC, TRG, primary immunodeficiency, newborn screening, aging

## Abstract

Quantification of T-cell receptor excision circles (TRECs) has impacted on human T-cell research, but interpretations on T-cell replication have been limited due to the lack of a genomic coding joint. We here overcome this limitation with multiplex TRG rearrangement quantification (detecting ~0.98 alleles per TCRαβ+ T cell) and the HSB-2 cell line with a retrovirally introduced TREC construct. We uncovered <5 cell divisions in naive and >10 cell divisions in effector memory T-cell subsets. Furthermore, we show that TREC dilution with age in healthy adults results mainly from increased T cell replication history. This proliferation was significantly increased in patients with predominantly antibody deficiency. Finally, Guthrie cards of neonates with Down syndrome have fewer T and B cells than controls, with similar T-cell and slightly higher B-cell replication. Thus, combined analysis of TRG coding joints and TREC signal joints can be utilized to quantify *in vivo* T-cell replication, and has direct applications for research into aging, immunodeficiency, and newborn screening.

## Introduction

Adaptive immunity is a critical component of the vertebrate immune system and is represented cellularly by B- and T-lymphocytes. Their crucial roles are illustrated in patients with inborn errors of immunity (IEI) (1). For example, patients with severe combined immunodeficiency (SCID) primarily lack mature T cells resulting in a lethal immunodeficiency if untreated (2). Predominantly antibody deficiency (PAD) is more common (3), and infectious complications in these patients can be managed with immunoglobulin replacement and prophylactic antibiotics. Still, about 68% of patients develop non-infectious complications, including autoimmunity and malignancies, which lead to high morbidity and early mortality (4–6). Hence, there is a need for early diagnosis of both severe and milder forms of IEI, as well as reliable markers that could predict future complications.

Similar to all blood cells, B and T lymphocytes are continuously produced throughout life. Progenitor B and T cells in bone marrow and thymus, respectively, generate unique antigen receptors through genomic rearrangements of their immunoglobulin (Ig) and T-cell receptor loci. In this process, coding joints are formed on chromosomes, and signal joints on circular excision products that are stably present in the cell, but are not replicated during cell divisions (7). Newly-formed T cells carry T-cell receptor excision circles (TRECs), whereas in memory T-cell populations these are extremely diluted following cell divisions. As such, TRECS are markers for thymic output (8). Indeed, PCR-based quantitative detection of TRECs has been applied to examine the effects of novel antiviral therapies on the thymic output in patients with HIV infection (8, 9), and following stem cell transplantation (10). Furthermore, TREC detection is currently utilized in many countries world-wide for newborn screening of SCID (11, 12).

More recently, we have introduced the use of Ig kappa deleting recombination excision circles (KRECs) of intronRSS-Kde rearrangements to examine B-cell replication (13). Analogous to TRECs, KREC quantification has been incorporated in several newborn screening protocols to detect absence of B cells for identification of X-linked agammaglobulinemia (XLA) and B-cell negative SCID cases (14, 15). The intronRSS-Kde coding joints remain stably present in the genome of mature B cells (16, 17). As a result, the ratio of these genomic coding joints to KREC signal joints is a direct measure for the average number of cell divisions a population of B cells has undergone (13). This accurate quantification has enabled delineation of T-cell dependent and – independent B-cell responses (18), as well as abnormal proliferation of B-cell subsets in common variable immunodeficiency (CVID; a form of PAD) (19) and Down syndrome (20).

In contrast to intronRSS-Kde coding joints, nearly all δREC-ψJα coding joints are removed from the genome in thymocytes during subsequent Vα-Jα gene rearrangements (21, 22). As a result, these cannot be used as a reliable marker for T-cell input, which complicates the use of TRECs to accurately determine T-cell replication history (23). We here present the means to overcome these limitations through the use of a multiplex PCR assay, which detects Vγ-Jγ gene rearrangement coding joints that are stably present in TCRαβ expressing T cells. Together with a newly generated TREC-containing cell line, these can be used to accurately quantify T-cell replication history. We describe accurate replication histories of naive and memory T cell subsets, enhanced T-cell replication with aging and abnormal T-cell replication in PAD patients. Finally, Vγ-Jγ and intronRSS-Kde coding joints can be reliably quantified from Guthrie cards and might form the basis of a second-tier test for absence of TRECs and/or KRECs in neonatal screening for IEI.

## Materials and Methods

### Research Subjects and Ethics

All studies were conducted in accordance with the declaration of Helsinki. Blood samples from adult patients with XLA or genetically-undefined PAD, as well as healthy adults were obtained after written informed consent was provided. Buffy coats were obtained from anonymous donors from the Australian Red Cross. These studies were approved by the human ethics committees of The Alfred Hospital (109/15) and Monash University (MEC# CF15/771 and 2016-0289).

Stored Guthrie cards of 107 Dutch anonymous controls and 84 children with Down syndrome, prepared ~3–5 days after birth, were collected after obtaining parental consent (24). All Guthrie cards had been stored at room temperature for 3–9 years. This study was approved by the Medical Ethical Committee “METOPP” employed by the Jeroen Bosch Hospital, the Netherlands.

### Generation of Control Cell Lines for the TREC-Assay

**Figure 1:**
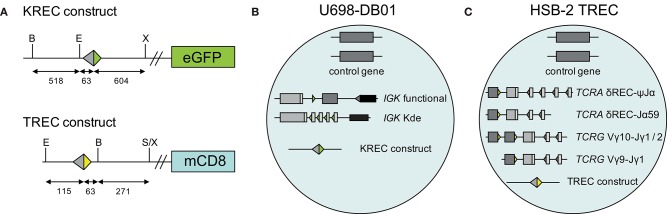
Generation of TREC signal joint containing cell lines. **(A)** Schematic overview of KREC and TREC constructs. Colored triangles depict RSS, fragment sizes (in bp) are depicted below the constructs, restriction sites: B, BamHI; E, EcoRI; S, SalI; X, XhoI. **(B)** Genetic composition of U698-DB01 and **(C)** HSB-2 TREC cell lines.

A human TREC signal joint was PCR amplified in two parts from genomic DNA of thymocytes to introduce a *Bam*HI restriction site 63 bp downstream of the signal joint, and subsequently cloned into the retroviral LZRS-IRES-lyt2 vector (Figure 1A). The LZRS-TREC construct was transfected into the Phoenix amphotropic packaging cell line using Fugene-6 (Roche Molecular Biochemicals, Branchbury, NY). Stable high-titer producer clones were selected with puromycin (1 μg/ml). The U698-DB01 pre-B-cell line (13, 25) and the HSB-2 immature T cell line (26) were cultured for several days in RPMI 1640 medium containing 10% FCS and antibiotics before transduction using Retronectin-coated Petri dishes (Takara, Shiga, Japan) and recombinant retrovirus containing supernatant for 2 days, with daily replenishing of retroviral supernatant. Cells expressing mouse CD8 (from the *lyt2* insert) were single-cell sorted using a FACSAriaI cell sorter (BD Biosciences). Individual clones were selected for dim mCD8 expression suggesting a single genomic integration, and subsequently subjected to real-time quantitative PCR to confirm the single-copy integration (see below).

### Isolation of T-Cell Subsets From Human Blood

Post-Ficoll mononuclear cells from blood bank donors were stored in 10% DMSO in liquid nitrogen prior to use. Using magnetic bead-based positive selection, CD4+ T cells were separated from thawed samples, followed by positive selection for CD8+ T cells (Dynabeads; Thermo Fisher). Both T-cell fractions were stained with fluorochrome-conjugated antibodies (Table S1) prior to sort-purification of four CD4+ and four CD8+ T-cell subsets on a FACSAriaI (BD Biosciences).

### DNA Extraction From Full Blood, Cell Lines, T-Cell Subsets, and Guthrie Cards

Genomic DNA was isolated from 200 μl whole blood of adult controls and antibody-deficient patients using a whole blood DNA extraction kit (Sigma-Aldrich) and eluted in 200 μl MilliQ. A genomic DNA Miniprep kit (Sigma-Aldrich) was used to isolate DNA from cultured cell lines and sort-purified T-cell subsets. DNA from 3 millimeter punches of Guthrie cards was isolated using the Sigma Genelute DNA Kit, according to the manufacturer's instructions and eluted in 100 μl MilliQ.

### Real-Time Quantitative PCR (RQ-PCR)

Independent RQ-PCR reactions were performed in duplicate for the albumin, TREC, KREC, intronRSS-Kde, ψJα_germline, and TRG assays. All experiments with whole blood and T-cell subset DNA were performed in a total mixture of 15 μl containing TaqMan GE Mastermix (Thermo Fisher Scientific), 540 nM of each primer (180 nM in case of multiplex mixtures), 60 nM of each 6-FAM/ZEN/Iowa Black labeled probes (Integrated DNA Technologies) and were run on the QuantStudio 6 Flex (Thermo Fisher Scientific). Five microliter of DNA eluate from Guthrie cards were run in RQ-PCR mixtures of 25 μl containing TaqMan Universal MasterMix (Applied Biosystems, Foster City, CA), 900 nM of each primer (300 nM in case of multiplex mixtures), 100 nM of each FAM-TAMRA labeled probe, 0.4 ng BSA, and were run on the StepOnePlus system (Life Technologies). The primers and probes are listed in Table S2. Total DNA input per reaction was generally between 30 and 200 ng and only samples with duplicates differing <1 Ct were included in the calculations.

### Calculations

The difference in Ct values between albumin and either the intronRSS-Kde and TRG coding joints or the intronRSS-Kde and δREC-ψJα signal joints were used to calculate the frequencies of cells carrying these rearrangements in unpurified leukocytes. To correct for any technical variation (efficiency) of the independent PCR reactions, the assays were run in parallel on the U698-DB01 and HSB-2 TREC cell lines. As the U698-DB01 cell line contains one intronRSS-Kde coding joint and one signal joint per genome (Figure 1B), and the HSB-2 cell line contains one δREC-ψJα signal joint per genome (Figure 1C), the frequency of cells in a sample containing these was calculated as follows:

$$\begin{array}{l} 2^{\left[{\left({\text{CT}}_{\text{albumin}}-{\text{CT}}_{\text{rearrangement}}\right)}_{\text{sample}}-{\left({\text{CT}}_{\text{albumin}}-{\text{CT}}_{\text{rearrangement}}\right)}_{\text{cell line}}\right]}\cdot 100\% \end{array}$$

Because the HSB-2 TREC cell line contains two TRG coding joints per genome (Figure 1C), the outcome of the equation above was multiplied by 2 to obtain the frequency of T cells rather than the frequency of rearranged TRG alleles per haplotype.

Absolute copy numbers were calculated with the assumption that the DNA content in human cells is 6.6 pg/cell (27, 28). We did not correct for the additional chromosome 21 in patients with Down syndrome (relative weight contribution <2%).

The difference in Ct values between the intronRSS-Kde coding and signal joints, and the TRG coding and TREC signal joints are directly related to the replication history of B cells and T cells, respectively. Taking into account the technical variation, the replication histories were calculated as follows:

$$\begin{array}{lll} \text{B}-\text{cell replication}: & \; & {\left({\text{CT}}_{\text{KREC}}-{\text{CT}}_{\text{intronRSS}-\text{Kde}}\right)}_{sample}\\ \; & - & {\left({\text{CT}}_{\text{KREC}}-{\text{CT}}_{\text{intronRSS}-\text{Kde}}\right)}_{\text{cell line}} \end{array}$$

and

$$\begin{array}{l} \text{T}-\text{cell replication}:\;{\left({\text{CT}}_{\text{TREC}}-{\text{CT}}_{\text{TRG}}\right)}_{sample}\\ \;-{\left({\text{CT}}_{\text{TREC}}-({\text{CT}}_{\text{TRG}}+1)\right)}_{cell\;line} \end{array}$$

As indicated, the formula to calculate T-cell replication corrects for the presence of 2 TRG alleles per genome vs. only 1 TREC allele per genome.

### Statistics

Statistical analyses were performed using the Mann-Whitney test for comparing unpaired samples and the Spearman R test for correlation analysis as indicated in the figure legends (GraphPad Prism 8.2.0 for Mac). Correlations were compared using Fisher r to z test. A *p* < 0.05 was considered statistically significant.

## Results

### Development of a Multiplex TRG Assay to Quantify T Cells in Blood

**Figure 2:**
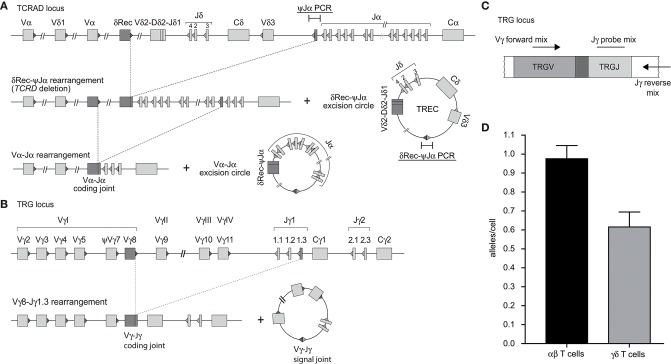
Development of a multiplex TRG PCR to quantify Vγ-Jγ coding joints as a marker for T cells. **(A)** Sequential rearrangements in the *TCRAD* locus. Following V(D)J recombination of *TCRD*, the whole locus is then deleted in the αβ+ T-cell lineage, predominantly by δREC–ψJα rearrangements. The rearrangements give rise to a δREC–ψJα signal joint on an excision circle (TREC) and a δREC–ψJα coding joint in the genome. The coding joint is deleted from the genome by TCRA (Vα-Jα) rearrangements and is then located on an excision circle as well (22, 23). **(B)** Rearrangements of TRG locus resulting in formation of Vγ-Jγ coding joint. **(C)** Schematic overview of the multiplex TGR PCR assay, which contains 4 Vγ forward primers, 2 Jγ reverse primers and 2 Jγ probes (See Table S2). **(D)** Mean number of rearranged *TRG* alleles per cell detected by multiplex TRG RQ-PCR assay in purified TCRαβ+ and TCRγδ+ T cells. The reduced detection in TCRγδ+ T cells is the result of our deliberate decision to omit detection of the frequently utilized Jγ1.2 gene in TCRγδ+ T cells.

In contrast to the intronRSS-Kde rearrangement in B cells, the δREC-ψJα rearrangement in T cells is not an end-stage rearrangement, since the coding joint is removed from the genome during subsequent Vα-Jα gene rearrangements (Figure 2A) (23). Consequently, it is not possible to use the δREC-ψJα rearrangement for T-cell quantification or to calculate T-cell proliferation in combination with TRECs. To overcome this limitation, we designed a multiplex RQ-PCR assay to amplify TRG gene rearrangements. This locus was chosen because it is rearranged in nearly all T-cell progenitors, it is both a one-step and an end-stage rearrangement (not deleted from the genome), and carries limited numbers of V and J genes (Figure 2B) (29, 30). In the design, detection of the Jγ1.2 gene was omitted as this is specifically used in TCRγδ-expressing T cells, which hardly undergo δREC-ψJα rearrangements (30).

Individual primer combinations were tested for similar efficiencies using genomic DNA from multiple T-cell lines that had rearranged distinct Vγ and Jγ genes (Table S3) (31). The final combination was tested on DNA from purified TCRαβ+ and TCRγδ+ T cells (Figures 2C,D). In line with previous observations (30), the ψJα gene was deleted in nearly all TCRαβ+ T cells, whereas it was still abundant in TCRγδ+ T cells. The new TRG assay detected 0.98 *TRG* gene rearrangements per TCRαβ+ T cell, whereas only 0.55 in TCRγδ+ T cells. Importantly, the *TRG* counts correlated significantly with absolute T-cell counts, and the intronRSS-Kde coding joints with absolute B-cell counts (Spearman r = 0.3473, *p* = 0.0070, and Spearman r = 0.4172, *p* = 0.0010, respectively; Figure S1).

### Cell Line Controls for TREC Analysis

In our previous studies, we introduced an intronRSS–Kde signal joint construct into the genome of the U698-M cell line using retroviral transduction (Figure 1B) (13). As this cell line already contained one intronRSS–Kde coding joint and two albumin gene copies per genome, it could be used as technical control for the ratio of coding joints and signal joints to study B-cell proliferation, as well as the quantification of B cells in a mixture population. To enable similar technical correction for studies using TRECs, we inserted a ψJα-δREC signal joint construct in the HSB-2 cell line that contains a ψJα-δREC coding joint as well as two Vγ-Jγ coding joints that were amplified by the TRG assay (Figures 1C, 2).

### T-Cell Replication in Healthy Controls

**Figure 3:**
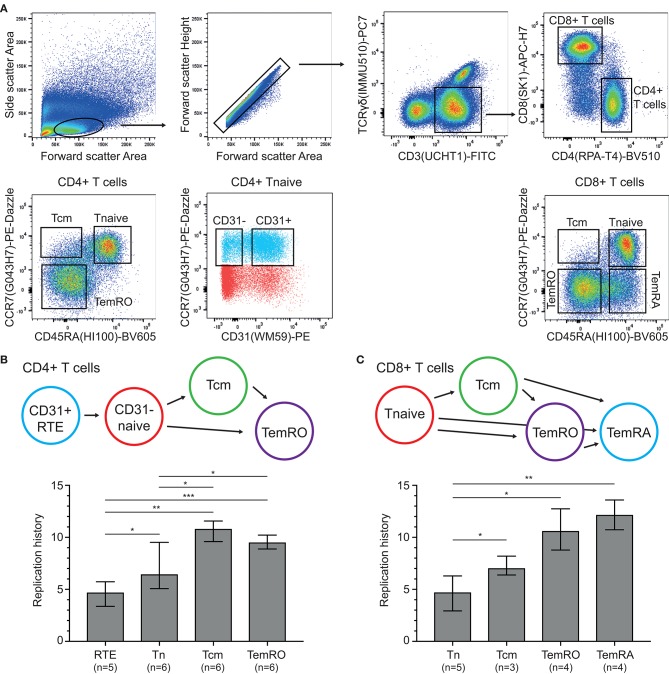
Replication histories of T-cell subsets in healthy controls. **(A)** Flow cytometric representation of T-cell differentiation stages. T-cell subsets were sort-purified based on the indicated gating strategy. **(B,C)** Schematic representation of CD4+ and CD8+ T-cell maturation and replication histories of the purified subsets. RTE, recent thymic emigrants; Tcm, central memory T cell; Tnaive, naive T cell; TemRO and TemRA, terminally differentiated effector memory T cell. The Mann-Whitney U test was used for statistical analysis: *p < 0.05; **p < 0.01; ***p < 0.001.

Having established the TRG assay and HSB-2 TREC control cell line, these were utilized to examine the replication histories of naive and memory/effector CD4+ and CD8+ T-cell subsets obtained from healthy controls (Figure 3A). Within CD4+ T cells, the CD31+ recent thymic emigrants (RTE) had undergone a median of 4.7 cell divisions and naive cells showed 6.5 cell divisions (Figure 3B). The antigen-experienced central memory (Tcm) and TemRO T cells, had increased levels, up to 10.8 and 9.5 cell divisions, respectively.

Naive CD8+ T cells showed a similar replication history as CD31+CD4+ naive T cells with 4.7 cell divisions (Figure 3C). These levels were significantly higher in Tcm cells with 7.1 cell divisions, and even more in the CCR7- TemRO and TemRA subsets with 10.6 and 12.2 cell divisions, respectively. Thus, in line with B-cell biology (13), the replication history of antigen-experienced T cells is significantly higher than in naive T-cell subsets.

### Contributions of Proliferation to Age-Associated Decline in TRECs

**Figure 4:**
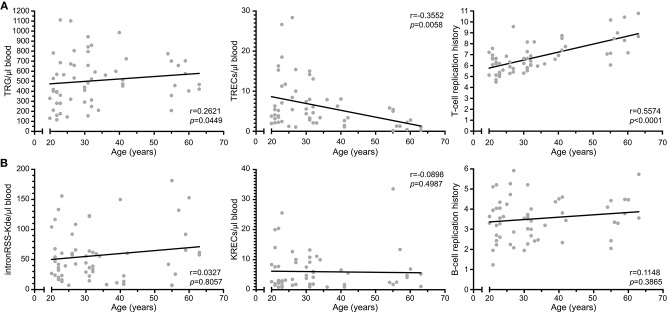
T-cell replication history increases with age. **(A)** Correlation plots of TRG, TRECs and T-cell replication histories as determined from whole blood vs. age of the donor. **(B)** Correlation plots of intronRSS-Kde, KREC and B-cell replication history. Data were obtained from 59 healthy controls. Spearman r was used for statistical analysis.

We examined TREC, TRG as well as the intronRSS-Kde and KREC assays in a cohort of 59 healthy controls (median age 31 years, range 20–63; Figure 4). Similar to Zubakov et al. (32), we found a significant decline in TRECs with age (r = −0.3552, *p* = 0.0058). The TRG copy numbers of controls increase with age (r = 0.2621, *p* = 0.0449), resulting in a significant increase of cell divisions with age (r = 0.5574, *p* < 0.0001).

The copy numbers of intronRSS-Kde, KRECs and B-cell replication history were not significantly correlated with age (Figure 4B). Thus, B-cell homeostasis changes minimally with aging, whereas the decline in TRECs with aging for the most part is the result of an increased T-cell replication history.

### Assessment of T-Cell Replication in Predominantly Antibody Deficiency

Quantification of TRECs and KRECs has been utilized before to subclassify patients with an antibody deficiency syndrome (33). As no coding joint assays were performed, it remains unclear what the contribution was of proliferation to the findings of abnormally low TRECs and KRECs. Therefore, we here applied our assays to 9 patients with genetically confirmed XLA and 42 patients with PAD (Table 1), who were subdivided in two phenotypical categories: infections-only (*n* = 14) and non-infectious complications (*n* = 28) (5, 6).

**Table 1:** Immunological and clinical characteristics of adults with inborn errors of immunity.

**Patient ID**	**Age at analysis (yr)**	**Sex**	**Gene**	**Mutation**	**B-cells^**#**^/μl blood**	**T-cells/μl blood**	**IgG at diagnosis (g/L)**	**IgA (g/L)**	**IgM (g/L)**	**Impaired vaccination response**	**Infectious complications**	**Non-infectious complications**
**X-LINKED AGAMMAGLOBULINEMIA (XLA)**												
XLA-01	18	M	BTK	c.1257delG	** <1**	*3,148*	N/A	N/A	N/A	N/A	None	None
XLA-02	21	M	BTK	c.1257delG	** <1**	1,652	N/A	**0.1**	**0.1**	N/A	Pneumonia, bronchiectasis	None
XLA-03	22	M	BTK	c.1257delG	** <1**	1,770	N/A	** <0.1**	** <0.1**	N/A	Otitis, sinusitis, pneumonia	None
XLA-04	24	M	BTK	c.1257delG	**10**	1,401	N/A	**0.1**	**0.1**	N/A	Sinusitis, bronchiectasis, prostatitis	None
XLA-05 *	24	M	BTK	c.1908+1G>C	** <0.1**	1,497	N/A	** <0.1**	** <0.1**	N/A	Otitis, sinusitis, pneumonia	Pre-B-ALL
XLA-06	26	M	BTK	c.1257delG	** <0.1**	2,478	N/A	** <0.1**	** <0.1**	N/A	Otitis, sinusitis, pneumonia, bronchiectasis	None
XLA-07	34	M	BTK	c.862C>T	**1**	**625**	N/A	** <0.1**	** <0.1**	N/A	Otitis, sinusitis, pneumonia	None
XLA-08	49	M	BTK	c.1559G>A	**3**	**801**	N/A	**0.1**	**0.1**	N/A	Otitis, sinusitis, pneumonia, bronchiectasis	None
XLA-09	59	M	BTK	c.1787+71C>T	** <0.1**	*3,141*	N/A	** <0.1**	** <0.1**	N/A	Otitis, sinusitis, pneumonia, asthma/COPD, bronchiectasis	None
**PAD**												
PAD-01	23	F	N/A	N/A	215	1,339	**4.4**	**0.2**	0.4	N/A	Otitis, sinusitis	Enteropathy
PAD-02	23	M	N/A	N/A	446	1,422	**4.9**	**0.3**	**0.3**	N/A	Sinusitis	ITP, AIHA, neutropenia, splenomegaly
PAD-03	24	F	N/A	N/A	**17**	**736**	** <1.4**	** <0.15**	** <0.2**	N/A	Pneumonia, sinusitis,	Arthritis, enteropathy
PAD-04	24	M	N/A	N/A	228	**954**	**1.1**	** <0.1**	** <0.1**	N/A	Pneumonia, otitis, sinusitis, VZV	None
PAD-05	25	M	N/A	N/A	230	1,381	**2.2**	** <0.1**	**0.1**	N/A	Sinusitis, pneumonia	Arthritis
PAD-06	26	M	N/A	N/A	467	*3,355*	N/A	**0.6**	**0.3**	Pneumococcal	Bronchitis, sinusitis, pneumonia	None
PAD-07	27	F	N/A	N/A	**166**	2,008	**3.9**	**0.7**	**0.3**	N/A	Pneumonia, bronchitis	Vitiligo
PAD-08	28	M	N/A	N/A	**37**	**700**	**3.3**	1	0.4	N/A	Bronchitis, pneumonia	Pulmonary nodules, colitis
PAD-09	29	F	N/A	N/A	**144**	1,982	**5.2**	**0.3**	0.5	Normal	Asthma/COPD	None
PAD-10	30	F	N/A	N/A	*1,089*	2,953	**2.2**	**0.4**	0.6	N/A	Sinusitis	Cytopenia
PAD-11	31	F	N/A	N/A	361	**983**	**5.6**	**0.3**	1.3	Normal	Sinusitis, systemic viral infection, giardia	Enteropathy
PAD-12	34	F	N/A	N/A	**46**	**1,083**	** <1**	** <0.1**	** <0.1**	N/A	Otitis, sinusitis, pneumonia, bronchiectasis	GLILD
PAD-13	35	F	N/A	N/A	222	**646**	**2.0**	**0.1**	**0.1**	Pneumococcal	Sinusitis, giardia, asthma/COPD	Enteropathy
PAD-14	37	M	N/A	N/A	**78**	**981**	**4.1**	**0.1**	0.4	Pneumococcal, Hib	Otitis, sinusitis, pneumonia, systemic viral infection, giardia, asthma/COPD, bronchiectasis	Granuloma, enteropathy
PAD-15	37	M	N/A	N/A	**1.3**	**901**	**2.1**	** <0.1**	** <0.1**	Pneumococcal	Pneumonia, asthma, bronchiectasis, chlamydia	ITP, eczema
PAD-16	40	F	N/A	N/A	202	1,038	**5.2**	1.5	0.9	Normal	Sinusitis, pneumonia	None
PAD-17	41	M	N/A	N/A	**35**	1,107	**2.6**	**0.2**	**0.2**	Pneumococcal	Sinusitis, pneumonia, systemic viral infection,	Granuloma, enteropathy
PAD-18	43	F	N/A	N/A	**101**	1,242	**2.0**	**0.3**	**0.2**	Pneumococcal	Sinusitis, pneumonia	Cytopenia
PAD-19	43	F	N/A	N/A	**116**	1,554	**4.2**	3.2	0.6	Pneumococcal	Sinusitis, pneumonia	Inflammatory tracheal stenosis
PAD-20	44	F	N/A	N/A	**177**	**614**	**2.7**	**0.1**	**0.1**	Pneumococcal	Sinusitis, pneumonia, giardia	Splenomegaly, enteropathy
PAD-21	44	M	N/A	N/A	**91**	**834**	**0.6**	**0.0**	**0.2**	N/A	Sinusitis, pneumonia, bronchiectasis	Splenomegaly, lymphadenopathy, granuloma, enteropathy, arthritis
PAD-22	45	M	N/A	N/A	N/A	N/A	**4.7**	2	1.2	N/A	None	HUS
PAD-23	46	M	N/A	N/A	228	**870**	**0.6**	**0.1**	**0.1**	Pneumococcal	Otitis, sinusitis	None
PAD-24	47	F	N/A	N/A	**84**	1,210	**3.1**	** <0.1**	** <0.1**	N/A	Sinusitis, bronchitis,	Colitis, autoimmunity
PAD-25	50	F	N/A	N/A	N/A	N/A	**5.8**	**0.3**	**0.2**	Pneumococcal	Otitis, sinusitis, pneumonia	None
PAD-26	52	F	N/A	N/A	**96**	**678**	**3.8**	0.9	0.8	N/A	Bronchitis, sinusitis	Pericarditis
PAD-27	52	F	N/A	N/A	193	1,329	**5.2**	**0.8**	1.3	pneumococcal	Sinusitis, pneumonia	None
PAD-28	52	M	N/A	N/A	195	**473**	**3.2**	**0.3**	**0.2**	Hib	Sinusitis, pneumonia	None
PAD-29	54	M	N/A	N/A	245	**775**	**2.9**	** <0.1**	**0.1**	N/A	Sinusitis, pneumonia, asthma/COPD, bronchiectasis	None
PAD-30	54	M	N/A	N/A	**152**	**1,040**	**4.4**	**0.2**	1.0	Pneumococcal, Hib, diphtheria, tetanus	Sinusitis, pneumonia	Solid organ malignancy
PAD-31	54	F	N/A	N/A	373	1,473	**5.4**	1.6	**0.3**	Pneumococcal	Bronchiectasis, sinusitis	Hypothyroidism
PAD-32	54	F	N/A	N/A	**95**	**631**	N/A	**0.2**	**0.4**	N/A	Viral pneumonia, otitis	None
PAD-33	55	F	N/A	N/A	215	1,362	**4.1**	**0.4**	0.8	Normal	Pneumonia	Inflammatory tenosynovitis
PAD-34	55	F	N/A	N/A	**138**	1,214	**4.8**	**0.8**	1.4	Pneumococcal	Otitis, sinusitis, pneumonia	None
PAD-35	56	M	N/A	N/A	**86**	**235**	N/A	** <0.1**	**0.2**	N/A	Pneumonia, hepatitis, colitis	AIHA, ITP, splenomegaly
PAD-36	60	F	N/A	N/A	**119**	1,408	**5.9**	**0.7**	**1.3**	N/A	Sinusitis, otitis, pneumonia	Hashimoto's thyroiditis
PAD-37	62	F	N/A	N/A	204	1,229	**5.6**	1.3	**0.3**	Pneumococcal	Sinusitis	None
PAD-38	66	F	N/A	N/A	**161**	**539**	**3.5**	**0.6**	**0.2**	N/A	Bronchitis, bronchiectasis, asthma	None
PAD-39	67	F	N/A	N/A	191	1,844	**4.6**	**0.6**	*2.9*	Diphtheria	Sinusitis, pneumonia, asthma/COPD	Enteropathy, autoimmunity
PAD-40	73	F	N/A	N/A	**154**	1,345	**3.3**	**0.3**	**0.3**	N/A	Sinusitis, asthma/COPD, bronchiectasis	None
PAD-41	77	F	N/A	N/A	**70**	**553**	**4.2**	**0.8**	1.9	Diphtheria	Systemic viral infection, bronchiectasis, ILD	Solid organ malignancy, auto-immunity
PAD-42	82	M	N/A	N/A	**13**	**727**	N/A	**0.6**	**0.2**	N/A	Otitis, sinusitis, pneumonia, bronchiectasis	Cytopenia
Normal range					190–550	1,090–3,020	6.10–16.2	0.85–4.99	0.35–2.42			

# Values of lymphocyte subsets and immunoglobulin levels below and above normal values are marked in bold and italic font, respectively. *Patient XLA-05 has been described before by van Zelm et al. (34).

*AIHA, auto-immune hemolytic anemia; COPD, chronic obstructive pulmonary disease; GLILD, granulomatous-lymphocytic interstitial lung disease; Hib, Haemophilus influenza B; HUS, hemolytic-uremic syndrome; ILD, interstitial lung disease; ITP, immune-mediated thrombocytopenia; VZV, varicella zoster virus; N/A, not available*.

**Figure 5:**
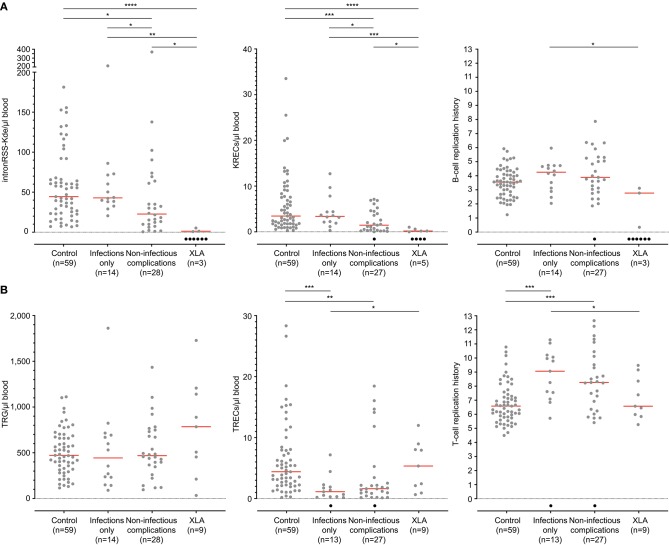
B-cell and T-cell replication histories in PAD patients. **(A)** intronRSS-Kde, KRECs and B-cell replication histories, and **(B)** TRG, TRECs and T-cell replication history for healthy controls and patients with XLA (*n* = 9) and PAD (CVID and hypogammaglobinemia) with infections only (*n* = 14) and non-infectious complications (*n* = 28) as determined from whole blood. Obtained values are shown in gray and undetectable values are shown in black; numbers indicated represent detectable values, and only they were included for statistical analysis with the Mann-Whitney *U*-test: *p < 0.05; **p < 0.01; ***p < 0.001; ****p < 0.0001.

IntronRSS-Kde and KREC copy numbers in patients with XLA were low or undetectable. The B-cell replication history could therefore only be calculated in 3 patients, which was decreased in one patient (0.3 cell divisions) and normal in the remaining two patients (2.8 and 3.1 cell divisions; Figure 5A). Of note, the absolute B-cell counts in these three patients were <1, <1, and 3 per microliter blood. Patients with PAD had normal copy numbers of intronRSS-Kde and lower numbers of KRECs (*p* = 0.0085). Also, their B-cell replication history was higher than in controls (median 4.063 vs. 3.550, *p* = 0.0268). Subsequently, we focused on patients with non-infectious complications and found lower KREC copy numbers as compared to patients with the infections-only phenotype (*p* = 0.0453). Compared to aging in healthy controls, patients with PAD show similar age-related correlations to T- and B-cell replication (Figure S2).

The studies of the T-cell compartment in patients with XLA showed normal TRG and TREC copy numbers, as well normal replication history (Figure 5B). For patients with PAD, normal TRG copy numbers, but decreased TREC counts and increased T-cell replication history were found as compared to controls (both *p* < 0.0001). There were no differences between patients with and without non-infectious complications.

### B- and T-Cell Replication in Dried Blood Spots of Children With Down Syndrome

**Figure 6:**
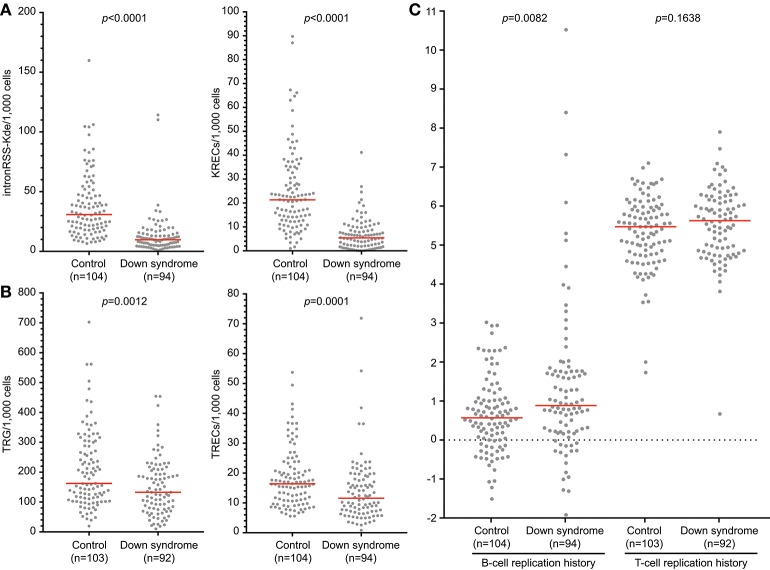
B-cell and T-cell replication histories in neonates with Down syndrome. IntronRSS-Kde and KREC **(A)**, and TRG and TREC **(B)** in healthy neonates and children with Down syndrome, corrected for DNA input as determined from Guthrie cards. **(C)** B- and T-cell replication histories for healthy neonates and children with Down syndrome. The Mann-Whitney *U*-test was used for statistical analysis.

Newborn screening for T-cell and B-cell lymphopenia is highly sensitive but has a poor specificity (35). Unfortunately, it is currently not possible to determine which patients should be referred urgently and for which patients a repeat screening test should be requested. Quantification of intronRSS-Kde and TRG copy numbers and calculation of the B- and T-cell replication history could potentially be part of second-tier testing in newborns with abnormal screening results to facilitate a risk assessment. In a previous study we showed that newborns with Down syndrome have lower numbers of KRECs and TRECs than healthy controls (24), which we replicated here (Figures 6A,B). Since DNA recovery from stored Guthrie cards is less predictable, we corrected our results for DNA input by including a control PCR targeting the albumin gene.

Children with Down syndrome have reduced intronRSS-Kde and TRG copy numbers compared to healthy newborns, which is consistent with previous studies showing decreased absolute B- and T-cell counts (Figures 6A,B) (36, 37). Subsequently, we calculated the replication histories. In healthy newborns, B cells have undergone a limited number of cell divisions (median 0.57) and T cells show ~5 cell divisions, which is consistent with results from naive B- and T-cell subsets (Figure 6C) (13). In comparison, newborns with Down syndrome showed a slightly increased number of cell divisions in their B-cell compartment than controls (0.885 vs. 0.57, *p* = 0.0082, Figure 6C). This could be the result of compensatory proliferation as a result of decreased bone marrow output or increased apoptosis (20, 38). The number of cell divisions for T cells were similar between children with Down syndrome and controls.

In addition to calculation of replication histories, the intron-RSS and TRG assays could be applied as a follow-up test in newborn screening settings to confirm or refute a positive finding with the KREC and TREC assays, respectively. We calculated the sensitivity and specificity of the intronRSS-Kde and TRG assays in our cohort of children with Down syndrome, assuming the KREC and TREC assays as the Gold standard and that values below the 5th percentile of controls were abnormal. Based on abnormal KREC results (“positive test results”), the sensitivity and specificity of the intronRSS-Kde assay were 75 and 80%, and for TRG to confirm an abnormal TREC result these were 45 and 97%. If used as a second tier test, the intron-RSS-Kde assay would confirm the abnormal KREC in 75% of cases, and the TRG assay would do so in 97% of abnormal TREC cases.

## Discussion

Here we demonstrate the development and application of a novel multiplex TRG RQ-PCR assay and our TREC containing T-cell line with biallelic TRG rearrangements enable the quantitative analysis of T-cell replication in normal immunobiology as well as immunodeficiency. By using this approach, we showed that naive T-cell subsets of healthy controls have undergone a limited number of cell divisions (<5) in contrast to antigen experienced effector T cells (>10). Furthermore, we showed that patients with PAD have an abnormal increase in both B- and T-cell replication history. By applying these assays on dried blood spot samples of healthy newborns, we showed that the intronRSS-Kde and TRG coding joint assays showed similar reductions in number as the TREC and KREC signal joint assays. Hence, the coding joint assays could have a role in second-tier testing after screening has identified abnormally decreased TREC and/or KREC levels.

We present a new approach for *ex vivo* quantification of *in vivo* T-cell replication histories. A variety of DNA labeling and intracellular lysine residues techniques have been used to study T-cell replication (39–41). Most previously reported approaches are limited to *in vitro* studies of human T-cell replication, whereas our RQ-PCR assay can be utilized to quantify the *in vivo* replication history. On the other hand, *in vivo* turnover of human T cells can be addressed by deuterium incorporation (42, 43). This has provided new insights into production and longevity of T cells. Ideally, *in vivo* deuterium labeling will be combined with *ex vivo* T-cell replication history analysis to obtain a full picture of T-cell production, longevity and replication in humans.

The TRG assay quantifies coding joints of Vγ-Jγ gene rearrangements and is therefore suitable as a means to quantify T-cell input. As δREC-ψJα coding joints are removed from the genome of developing T cells by Vα-Jα gene rearrangements, these cannot be utilized as a genomic marker (23). Previous studies have used other means to overcome this limitation of the “missing coding joint”. The most notable are those in which T cells or subsets have been FACS purified and a general genomic marker was chosen for TREC quantification (e.g., albumin, TRAC or CD3G) (9, 44, 45). Limitations for such an approach are the fact that each cell will contain 2 copies of these genomic markers, whereas δREC-ψJα gene rearrangements are found in less than half of TRA/D loci (30), with the remainder of the TRD loci being deleted by rearrangements involving other genetic elements (22). Moreover, sort-purification of T cells can restrict large-scale analysis of samples as it is labor intensive, costly and requires high cell numbers to yield sufficient material for RQ-PCR. The TRG assay being T-cell specific and detecting just under 1 allele/cell in αβT cells, i.e., very similar to the frequency of δREC-ψJα gene rearrangements. Finally, the combined use with our control cell line enables technical correction for PCR efficiencies, which is needed to accurately determine cell divisions.

As δREC-ψJα gene rearrangements are initiated relatively late during thymocyte development, TREC measurement includes the combined replication history of double positive and single positive CD4 thymocytes, as well as homeostatic proliferation in the periphery, but most likely not that of double negative thymocytes prior to TCRβ selection (30, 44, 45). With the TRG-TREC approach, we quantified the replication history of naive Th cells to be ~5 cell divisions for the CD31+ subset and ~6 for the CD31- subset. This is in line with the ~6 cell divisions for naive CD4+ T cells from young adults previously reported (44). In addition, it confirms earlier observations that the CD31+ subset is more enriched for TRECs and is likely enriched for RTEs (46, 47).

The replication histories of naive CD8+ and CD4+ T cells were very similar. In contrast, CD8+ Tcm had a much lower average replication history than CD4+ Tcm. Hence, despite their phenotypic similarity, this indicates that different processes underlie their generation, i.e., with distinct levels of proliferation. Alternatively, the CD8+ Tcm subset could be a mixture of memory cells with more naive T cells that have not undergone antigen-induced proliferation.

With normal aging, the total T-cell compartment remains stable in size while naive subsets decrease and effector populations increase (48, 49). This is reflected by stable TRG copy numbers throughout life and decreasing TRECs with higher age (32). As a result, the overall T-cell replication history increases with age.

In our study, we have focused on immunodeficiencies that are characterized by impaired antibody formation. Patients with XLA have strongly decreased or undetectable intronRSS-Kde copy numbers as well as KRECs. We calculated normal B-cell replication histories for 2 out of 3 patients, which is in line with previous findings (50). Normal T-cell replication history was found in patients with XLA. This is consistent with the fact that the BTK protein is normally not expressed in T cells of healthy controls, and T-cell biology does not appear to be affected (51).

Patients with PAD are heterogeneous with regards to their clinical presentations as well as immunologic investigations. These conditions are classically thought to be primarily caused by defective B-cell function. Nonetheless, a significant portion of patients are found to have T-cell abnormalities, especially showing decreased naive T-cell subsets (6, 52, 53). A study from Kamae et al. showed that patients with CVID who have decreased TRECs and KRECs are more likely to develop disease related complications (i.e., infections, autoimmune diseases and malignancies), warranting to consider a diagnosis of combined immunodeficiency (CID) (33). Here, we have shown alterations in the T-cell compartment of patients with PAD that confirm that not only B-cell immunity is affected. Currently, our methods do not allow for classification of patients with PAD in order to identify an underlying (genetic) cause or help to assess the risk for developing non-infectious complications. However, studying the replication history in sorted T-cell subsets might give more insight in the pathways that are affected in patients with PAD and allow for new classification strategies that will assist in clinical management of these patients. As the T-cell abnormalities were not found in XLA patients, it is suggestive that in at least a subset of PAD patients T-cell replication is affected intrinsically, or as a result of additional inflammatory effects not present in XLA patients.

We also demonstrated the potential to quantify the replication history of T-cell subsets. In future studies, this could provide new insights into Th-cell function. For example, Th17 cells which have an important role in the etiology of a variety of inflammatory conditions, including rheumatoid arthritis and inflammatory bowel disease (54, 55). Furthermore, the effects of immunosuppressive treatment could be studied to determine whether normal states can be achieved and serve as a disease monitoring feature. It should be noted that the calculation of T-cell replication will be limited in several disease settings. In cases of severe T-cell lymphopenia, the TREC and/or TRG potentially will not generate a signal, similar to what we observed for the KREC and intronRSS-Kde assays in our patients with XLA. Furthermore, in extreme T-cell lymphoproliferations, TRECs might be extremely diluted and unable to be detected with the TREC assay. This will be especially true in case of monoclonal proliferations, but potentially also in extreme polyclonal proliferations, in parallel to the dilution of KRECs in persistent polyclonal B-cell lymphocytosis (PPBL) (56).

Although newborn screening for T-cell and B-cell lymphopenia is highly effective, at least 80% of newborns with a positive test (reduced TRECs and/or KRECs) turn out to be “false positives” and do not have SCID or XLA. Currently, all abnormal results require drawing of a second blood sample for immunophenotyping. Ideally, a large number of false positives are identified using a second-tier DNA-based test. For example, 22q11 microdeletion syndromes and Down syndrome can be identified in a reliable manner via dried blood spot samples (24, 57). We were able to establish that our intronRSS-Kde and TRG assays generate reliable results on dried blood spot samples. These assays could be applied to samples from children with abnormal TREC and/or KREC screening results, prior to alarming their parents to request a second sample for additional testing.

We here described the development of a novel combined TRG coding joint RQ-PCR and δREC ψJα signal joint RQ-PCR assay with a TREC control cell line, which together allow reliable quantification of *in vivo* T-cell replication history. Our approach has led to new insights in normal T-cell biology, aging and immunodeficiency. Moreover, the high specificity of the TRG and intronRSS-Kde assays to confirm an abnormal TREC and KREC finding in neonates with Down syndrome shows a promise for application of these assays as second tier test in newborn screening. Still, extensive evaluation on large cohorts of neonates with genetically-confirmed SCID or XLA will be required to evaluate if application of these assays could reduce the number of cases in which a second blood sample for verification of abnormal results is required.

## Data Availability

The datasets generated for this study are available on request to the corresponding author.

## Author Contributions

MvZ and JvD conceptualized the study and designed experiments. RV, PA, EW, SD, and SB performed experiments. RV and MvZ analyzed and interpreted all data and wrote the manuscript. JB, PC, RS, and EdV established the patient sample collection protocols and included patients into the study. All authors commented on manuscript drafts and approved the final version.

### Conflict of Interest Statement

The authors declare that the research was conducted in the absence of any commercial or financial relationships that could be construed as a potential conflict of interest.
