# Supplementary material for: Quantification of T-Cell and B-Cell Replication History in Aging, Immunodeficiency, and Newborn Screening
Source: Front Immunol. 2019 Aug 29;10:2084. doi: 10.3389/fimmu.2019.02084 (PMC6730487; doi:10.3389/fimmu.2019.02084)
Supplement: Supplementary file 1 [file Table_1.DOCX]

**Supplemental Tables (n=3) and Figure legends (n=2)**

**Table S1.** **Antibodies used for cell sorting**

| **Antibody** | **Fluorochrome** | **Clone** | **Supplier** |
| --- | --- | --- | --- |
| CD3 | FITC | UCHT1 | BD Biosciences, San Jose, CA, USA |
| CD4 | BV510 | RPA-T4 | BioLegend, San Diego, CA, USA |
| CD8 | APC-H7 | SK1 | BD Biosciences |
| CD31 | PE | WM59 | BD Biosciences |
| CD45RA | BV605 | HI100 | BioLegend |
| CCR7 | PE-Dazzle | G043H7 | BioLegend |
| TCRγδ | PC7 | IMMU510 | Beckman-Coulter, Indianapolis, IN |

**Table S2.** Oligonucleotide sequences for RQ-PCR assays

| **Assay** | **Forward Primer(s) (5'-3')** | **Reverse Primer(s) (5'-3')** | **TaqMan Probe(s) (5'-3')** | **amplicon**  **size (bp)** |
| --- | --- | --- | --- | --- |
| albumin | TGAACAGGCGACCATGCTT ^a^ | CTCTCCTTCTCAGAAAGTGTGCATAT ^b^ | TGCTGAAACATTCACCTTCCATGCAGA ^b^ | 118 |
| KREC ^c^ | TCAGCGCCCATTACGTTTCT | GTGAGGGACACGCAGCC | CCAGCTCTTACCCTAGAGTTTCTGCACGG | 148 |
| intronRSS-Kde ^c^ | CCCGATTAATGCTGCCGTAG | CCTAGGGAGCAGGGAGGCTT | AGCTGCATTTTTGCCATATCCACTATTTGGAGT | 144 |
| TREC ^d^ | CCATGCTGACACCTCTGGTT | TCGTGAGAACGGTGAATGAAG | CACGGTGATGCATAGGCACCTGC | 131 |
| ψJα_germline ^30^ | TTTCAACCATGCTGACACCT | GGCACATTAGAATCTCTCACTGA | CTGACATTTGGAGCCAACACTAGAGGAATC | 153 |
| TRG | Vγ1f: GGAAGGCCCCACAGCRTCTT ^f^  Vγ9: CGGCACTGTCAGAAAGGAATC ^f^  Vγ10: AGCATGGGTAAGACAAGCAA ^f^  Vγ11: TGCTCACACTTCCACTTCCACTT | Jγ1.1-2.1: CATCTATCAGTTTTTCATTACTGGAATAA  Jγ1.3-2.3: TTCCTGCCTTCCCTCTATTACCT ^g^ | Jγ1.1-2.1: ACCAGGTGAAGTTACTATGAGCTTAGTCCCTTCAG  Jγ1.3-2.3: TGTCACAGGTAAGTATCGGAAGAATACAACATTTCC ^g^ | ~200-290 |
| a. (van Zelm et al., 2011)  b. (Pongers-Willemse et al., 1998)  c. (van Zelm et al., 2007)  d. (Hazenberg et al., 2000)  e. (Dik et al., 2005)  f. (van Dongen et al., 2003)  g. (van der Velden et al., 2002) | | | | |

**Table S3.** ***TRG* genes recognized by TRG assay primers**

| **Primer** | **TRG gene(s)** | **Reference** |
| --- | --- | --- |
| Vγ1f | TRGV2, TRGV3, TRGV4, TRGV5, TRGV8 | (van Dongen et al., 2003) |
| Vγ9 | TRGV9 | (van Dongen et al., 2003) |
| Vγ10 | TRGV10 | (van Dongen et al., 2003) |
| Vγ11 | TRGV11 |  |
| Jγ1.1-2.1 | TRGJ1, TRGJ2 | (van der Velden et al., 2002) |
| Jγ1.3-2.3 | TRGJ1P, TRGJ2P | (van der Velden et al., 2002) |

**Figure S1. Correlation between RQ-PCR and flow cytometry-based quantification of lymphocytes.**

(**A**) Correlation between rearranged TRG genes as quantified by multiplex PCR assay and absolute numbers of T cells/microliter blood as determined by flow cytometry.

(**B**) Correlation between rearranged intronRSS and Kde elements by RQ-PCR and absolute numbers of B cells/microliter blood as determined by flow cytometry. Correlations were calculated using Spearman r.

**Figure S2. Correlations between T- and B-cell replication and age.**

Correlation of T-cell replication history (**A**) and B-cell replication history (**B**) with age for healthy controls (grey) and patients with PAD (black). PAD, predominantly antibody deficiency (CVID and hypogammaglobulinemia). Correlations were calculated using Spearman r. No significant differences between both groups were found using Fisher r to z.
